# How Localized Z-Disc Damage Affects Force Generation and Gene Expression in Cardiomyocytes

**DOI:** 10.3390/bioengineering8120213

**Published:** 2021-12-14

**Authors:** Dominik Müller, Sören Donath, Emanuel Georg Brückner, Santoshi Biswanath Devadas, Fiene Daniel, Lara Gentemann, Robert Zweigerdt, Alexander Heisterkamp, Stefan Michael Klaus Kalies

**Affiliations:** 1 Institute of Quantum Optics, Leibniz University Hannover, 30167 Hannover, Germany; d.mueller@iqo.uni-hannover.de (D.M.); donath@iqo.uni-hannover.de (S.D.); emanuel.brueckner@stud.uni-hannover.de (E.G.B.); Fiene.Daniel@stud.mh-hannover.de (F.D.); Lara.Gentemann@stud.mh-hannover.de (L.G.); heisterkamp@iqo.uni-hannover.de (A.H.); 2 REBIRTH Research Center for Translational Regenerative Medicine, Hannover Medical School, 30625 Hannover, Germany; Devadas.Santoshi@mh-hannover.de (S.B.D.); zweigerdt.robert@mh-hannover.de (R.Z.); 3 Lower Saxony Centre for Biomedical Engineering and Implant Research and Development (NIFE), 30625 Hannover, Germany; 4 Leibniz Research Laboratories for Biotechnology and Artificial Organs (LEBAO), Department of Cardiac, Thoracic, Transplantation and Vascular Surgery (HTTG), Hannover Medical School, 30625 Hannover, Germany

**Keywords:** femtosecond laser manipulation, cardiomyocyte, Z-disc, traction forces

## Abstract

The proper function of cardiomyocytes (CMs) is highly related to the Z-disc, which has a pivotal role in orchestrating the sarcomeric cytoskeletal function. To better understand Z-disc related cardiomyopathies, novel models of Z-disc damage have to be developed. Human pluripotent stem cell (hPSC)-derived CMs can serve as an in vitro model to better understand the sarcomeric cytoskeleton. A femtosecond laser system can be applied for localized and defined damage application within cells as single Z-discs can be removed. We have investigated the changes in force generation via traction force microscopy, and in gene expression after Z-disc manipulation in hPSC-derived CMs. We observed a significant weakening of force generation after removal of a Z-disc. However, no significant changes of the number of contractions after manipulation were detected. The stress related gene NF-kB was significantly upregulated. Additionally, α-actinin (*ACTN2*) and filamin-C (*FLNc*) were upregulated, pointing to remodeling of the Z-disc and the sarcomeric cytoskeleton. Ultimately, cardiac troponin I (*TNNI3*) and cardiac muscle troponin T (*TNNT2)* were significantly downregulated. Our results allow a better understanding of transcriptional coupling of Z-disc damage and the relation of damage to force generation and can therefore finally pave the way to novel therapies of sarcomeric disorders.

## 1. Introduction

The proper function of the sarcomeric cytoskeleton is pivotal for cardiomyocyte (CM) contraction. It enables mechanotransduction and mechanosensation and has to adapt to physical activity, growth, or pathological stimuli [1,2]. During cardiac diastole, for instance, the sarcomere increases in length, which leads to a higher calcium sensitivity and increased force development during contraction [3]. Failure of remodeling the cytoskeletal architecture would lead to loss of homeostasis in the dynamic mechanical environment of the heart [1]. Consequently, several cardiomyopathies are associated with sarcomere dysfunction, for example, hypertrophic and dilated cardiomyopathies [2,4,5,6,7,8]. A major component of the sarcomeric cytoskeleton are Z-discs. They are the lateral boundaries of each sarcomere and central points for mechanical stability, force transmission, and signaling [9,10,11]. The proper structure of each Z-disc and the Z-disc connections are mandatory to maintain sarcomere function. The Z-disc is composed of hundreds of proteins, including its major component α-actinin [10,12,13]. These are involved in Z-disc assembly, regulation of contraction, stretch sensing and protein phosphorylation [4,11]. Z-disc proteins can also shuttle between the Z-disc complex and the nucleus in CMs and contribute to Z-disc transcriptional coupling. This includes, for instance, the proteins telethonin, muscle LIM protein (MLP), or FHL2 [14,15,16,17,18]. To better understand the role of Z-discs in orchestrating sarcomeric cytoskeletal function, novel models have to be conceived, which also investigate potential damage and repair mechanisms.

The directed cardiomyogenic differentiation of human pluripotent stem cells (hPSC), including both human embryonic (hESC) and induced pluripotent stem cells (hiPSC), enabled the reliable in vitro generation of human CMs [19]. These now present a promising approach for disease modeling, drug screening and regenerative biology. hPSC-derived CMs (hPSC-CM) can be efficiently generated in large quantities and can be well characterized in vitro [20]. It is well established that hPSC-CMs form well-organized sarcomeric structures when seeded on tissue culture surfaces [21,22,23]. In contrast to tissue-derived primary cells, hPSC-derived CMs are not connected to isolation artifacts and can, in particular when derived from patient specific hiPSC lines, represent a very promising strategy in modeling cardiomyopathies [24,25]. Several cardiomyopathies, in particular cardiac channelopathies have been investigated using hPSC-derived CMs, for example, long QT syndrome or catecholaminergic polymorphic ventricular tachycardia [24,26,27]. hPSC-derived CMs are different to neonatal derived CMs: they present different transcriptional profiles, have a more immature sarcomere organization similar to embryonic CMs, and lower contractile performance [23,28,29]. The electrophysiological properties are similar to those of neonatal cardiac myocytes [28]. On the one hand, the immature, embryonic myocyte-like sarcomere organization partly challenges the applicability for modeling sarcomere function in CMs. On the other hand, it provides an interesting feature reflecting the remarkable regenerative capacity of the mammalian embryonic heart in vitro [28]. Therefore, in vitro studies on hPSC-CMs may reveal valuable novel pathways involved in sarcomere repair in myocardial cells.

Another challenge in studying sarcomere remodeling and repair is to address single Z-discs in a cardiac cell. Gene knock-out or knock-down models, for example, do not allow to specifically investigate the role of single Z-discs. In contrast, we have recently developed a method to study single Z-disc damage in individual hPSC-CMs. A femtosecond laser is applied in combination with fluorescence imaging techniques to study ablation of single or multiple Z-discs [30,31].

**Figure 1:**
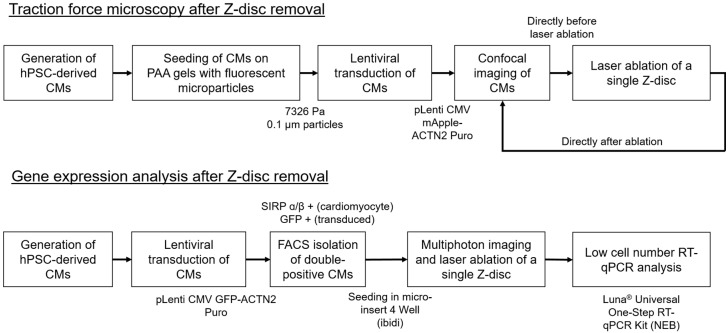
We have developed a workflow to investigate cellular traction forces and gene expression after ablation of a single Z-disc in cardiomyocytes despite low cell numbers.

The femtosecond laser induces multiphoton ionization in water and biomolecules, which leads to the formation of a so-called low-density plasma and bond-breaking of biomolecules [32]. Due to the ultrashort pulse length and the nonlinearity of the process, a high precision in nanosurgery can be reached, while thermal effects are negligible. While we could reveal kinetics of Z-discs repair, including time, structural changes, and calcium homeostasis, we were previously not able to investigate changes in force generation or the impact of transcriptional profiles. Consequently, we are addressing these challenges in the current study. Towards this aim, we have developed two methodologies (see Figure 1), which allow us to study gene expression based on low cell numbers and to use traction force microscopy to study force generation in single hPSC-CMs after ablation of a Z-disc.

## 2. Materials and Methods

### 2.1. Generation of hPSC-Derived Cardiomyocytes

hPSC-CMs were generated by directed differentiation according to Kempf et al. [19]. In brief, hPSCs were grown on GelTrex (Thermo Fisher Scientific, Darmstadt, Germany) in E8 medium. Cells were harvested using 40 µL/cm² Accutase for 5 min at 37 °C and inoculated as single cells at 1.5 × 10^5^ cells/mL in E8 supplemented with 10 µM Y-27632 (Tocris, Bristol, UK) in single use 125 mL Erlenmeyer flasks (Corning, Amsterdam, The Netherlands; working volume 20, mL) for 48 h in suspension culture on an orbital shaker (Celltron, Einsbach, Germany) at 70 revolutions per minute (rpm) and incubated at 37 °C and 5% CO_2_ for aggregate formation. For differentiation induction, the E8 medium was changed to CDM3 [33] supplemented with 5 µM CHIR99021 (Sigma-Aldrich, Taufkirchen, Germany) for precisely 24 h; fresh medium supplemented with 5 µM IWP2 (Tocris) was added thereafter and cells were cultured for 48 h before the medium was changed to fresh CDM3. Subsequently, the medium was refreshed every 2–3 days. The induced hPSC-CM content was analyzed by flow cytometry on MACSQuant Analyzer 10 (Miltenyi Biotec, Bergisch Gladbach, Germany) using FlowJo software (Flowjo LLC, Ashland, OR, USA) with antibodies specific to anti-sarcomere-specific pan-MyHC antibody (1:20, MF 20, Hybridoma Bank, Iowa City, IA, USA), anti-cardiac Troponin T (1:200, clone 13–11, Thermo Fisher Scientific, Germany), anti-sarcomeric α-actinin (1:800, EA53, Sigma-Aldrich, Germany) and respective anti-IgG isotype controls (DAKO), followed by an incubation with CyTM5-conjugated secondary antibody (1:200, Jackson ImmunoResearch, Ely, UK). Typically, an hPSC-CMs content of 85–95% was achieved when analyzed on day 10–14 of differentiation.

### 2.2. Polyacrylamide Gel Preparation and Traction Force Measurements

All experiments were performed using a Young’s modulus of 7326 Pa, which was validated via rotational viscosimetry using an MCR 302 Modular Rheometer (Anton Paar, Graz, Austria) at the Institute of Technical Chemistry (LUH Hannover, Hannover, Germany). For polyacrylamide (PAA) gel preparation, 200 µL stock solution (resulting in final concentration of 7.5% acrylamide and 0.3% N,N′-methylenebisacrylamide), 291.75 µL water, 5 µL 0.1 nm FluoSpheres™ yellow/green (Fisher Scientific, Schwerte, Germany), 0.75 µL N,N,N′,N′-tetramethylethylenediamine (TEMED) and 2.5 µL of 10% ammonium persulfate (Sigma-Aldrich, Germany) were mixed as described [34]. Twelve microliters of the acrylamide/bisacrylamide working solution were applied on activated 35 mm glass bottom dishes (Ibidi, Graefelfing, Germany) and squeezed by placing a 12 mm coverslip (coated with Sigmacote^®^, Sigma-Aldrich, Germany) on top of the droplet. Glass bottom dishes were activated beforehand via 2% (3-Aminopropyl)triethoxysilane in isopropanol and 1% glutaraldehyde (both Sigma-Aldrich, Germany) treatment, with several wash steps with water in between, to guarantee PAA gel adhesion to the glass surface [34].

After polymerization for 20 min, the coverslip was removed and the gel surface was functionalized using Sulfo-SANPAH (2 mg/mL, Hölzel Diagnostika Handels GmbH, Cologne, Germany). After UV irradiation for 10 min (wavelength 254 nm, power 6 W, distance 5 cm) the gel was washed with water and coated with a mixture of poly D-lysine 1 mg/mL and 2 µg/mL rat tail collagen (Sigma-Aldrich, Germany) for 2 h at room temperature. After washing three times with phosphate buffered saline (DPBS), gels were incubated for 30 min with cell medium prior to cell seeding. A total of 80,000 cells were seeded on each gel. The “CM support medium” (IMDM+GlutaMAX™ (Gibco) supplemented with 20% FCS (HyClone), 1 mM L-glutamine, 0.1 mM ß-mercaptoethanol (all Life Technologies, Darmstadt, Germany), 10 µM Y-27632, 1% non-essential amino acids, and 1% penicillin/streptomycin (Merck Millipore, Darmstadt, Germany)) was changed to RPMI 1640 (2 mM Glutamine) supplemented with B27 plus insulin (referred to as “RB+”) the following day. Seeded cells were transduced using a lentivirus pLenti CMV mApple-ACTN2 Puro to visualize Z-discs in cells 4 days after seeding. The vector was kindly provided by Christopher S. Chen (Department of Biomedical Engineering, Boston University, Boston, MA, USA) [35]. Lentivirus was produced as described previously [30,31]. Experiments were performed 5 days after transduction.

### 2.3. Traction Force Imaging and Laser Manipulation

CMs on PAA gels were imaged using a commercial inverted confocal setup Leica TCS SP5. In each experiment, microparticles, Z-disc pattern, and cell outline (transmission photomultiplier tube) were detected. During imaging, cells were incubated at 37 °C and 5% CO_2_ atmosphere using a custom build incubation chamber. The cell position in the confocal setup was saved using a motorized stage. For each measured cell, a video of several contractions was recorded before the cell dish was transferred to the laser system for Z-disc removal. A framerate of four FPS and 512 × 512 px with 0.12 px/µm were used.

To remove a single Z-disc, a separate Ti:Sapphire laser system with a pulse length of 140 fs and a repetition rate of 80 MHz was used in combination with multiphoton imaging and fluorescence microscopy [31]. The laser power entering the microscope was adjusted to guarantee equal experimental settings before each experiment. Additionally, the stage of both confocal microscope and ablation system were synchronized concerning the absolute positions using a custom build calibration slide. Both systems were in connected rooms to minimize transport. Z-discs in the cells were randomly selected and ablated at a wavelength of 730 nm as described in our previous studies [30,31]. During cell manipulation, cells were incubated at 37 °C and 5% CO_2_ atmosphere in a microscope incubation chamber (Okolab, Pozzuoli, Italy). Subsequently, the cell dish was transferred back to the confocal imaging setup and another video of several contractions was recorded using the same parameters.

For image analysis, videos were cut such that each contraction yielded a separate image series. All contractions per cell were analyzed before and after Z-disc removal using Fiji in combination with the iterative PIV plugin and the FTTC plugin [36,37,38]. A custom written macro used a frame of the cell in the relaxed state to analyze each contraction and to calculate the traction forces for each contraction time series. For each cell, the force during contraction and relaxation was averaged over all contractions. The cell shape was also extracted from every image and, based on the cell shape, the force per cell was calculated. In total, 16 CMs were analyzed.

### 2.4. Preparation of Cardiomyocytes for RT-qPCR Analysis

After dissociation of suspension culture derived hPSC-CM aggregates with the STEMdiff Cardiomyocyte Dissociation Kit (Stemcell Technologies, Cologne, Germany), one million hPSC-CMs per well were seeded in six well plates coated with 0.1% gelatin, and incubated with CM support medium at 37 °C and 5% CO_2_. The medium was changed to RB+ the next day and CMs were transduced with a multiplicity of infection (MOI) of one with pLenti CMV GFP-ACTN2 Puro lentiviral particles. The pLenti CMV GFP-ACTN2 Puro vector was kindly provided by Christopher S. Chen (Department of Biomedical Engineering, Boston University, Boston, MA, USA) [35]. After 3 days of CM cultivation, cells were washed with DPBS, harvested by using 40 µL/cm² Accutase for 5 min at 37 °C and pelleted by centrifugation at 700× *g*. CMs were resuspended with DPBS + 10% fetal bovine serum (FBS, PAN-Biotech, Aidenbach, Germany) and incubated with 10 µL PE/Cyanine7 anti-human CD172a/b (SIRPα/β) antibody (Biolegend, San Diego, CA, USA) for 20 min at 4 °C. After centrifugation, CMs were resuspended with DPBS + 10% FBS and 1 mM EDTA and filtered through a 70 µm cell strainer (pluriSelect Life Science, Leipzig, Germany). CMs were stored on ice until sorting for Cy7 and GFP positive cells on a FACSAria III Fusion cell sorter (BD Biosciences, Franklin Lakes, NJ, USA). For later laser treatment of CMs, four-well silicone micro-inserts (Ibidi, Germany) were inserted into 35 mm glass bottom dishes (Ibidi, Germany) and coated with collagen-poly-l-lysin as described above. Double positive CMs were resuspended in CM support medium and seeded into these micro-inserts. The medium was changed the next day to RB+ and RT-qPCR experiments were performed after 2 days incubation at 37 °C and 5% CO_2_ atmosphere.

### 2.5. RNA Extraction and Quantitative Real-Time Polymerase Chain Reaction (RT-qPCR)

For RT-qPCR experiments, all CMs in one of the four-well micro-inserts were treated by ablating a single Z-disc per CM. Untreated CMs in adjacent wells served as controls. Four hours after laser treatment of CMs, all cells (treated or control) per micro-insert well were lysed using the Luna Cell Ready Lysis Module (New England Biolabs, Frankfurt am Main, Germany). RT-qPCR was performed in duplicates with the Luna Universal One-Step RT-qPCR Kit (NEB, Germany). A TOptical thermocycler (Analytik Jena, Jena, Germany) was used for RT-qPCR with the following protocol: 55 °C for 10 min (RT), 95 °C for 1 min (initial denaturation), 45 cycles: 95 °C for 10 s, 60 °C for 30 s (amplification), melt curve in the range from 55 °C to 95 °C at 0.2 °C/s. A reaction volume of 10 µL with 1 µL cell lysate and 10 µM primers was used. All data were analyzed for relative gene expression as described by Taylor et al. [39]. Expression levels of target genes were normalized to ATP5F1 and GAPDH gene expression and related to the expression without ablation (calibrator CT). Primer sequences and analyzed genes are listed in the Appendix A. 

### 2.6. Data Analysis and Statistics

All data sets were analyzed and graphically represented using Origin (OriginPro 2018b, OriginLab, Northampton, MA, USA). Traction force data were normal distributed (*p* ≥ 0.16, Kolmogorov–Smirnov goodness of fit test) and analyzed via a two-sided Student *t*-test. In RT-qPCR experiments, a two-sided Welch corrected *t*-test was applied to account for unequal variances. *p* values of < 0.1 were considered as significant. Box plots are characterized by upper line of box, 75th percentile; lower line of box, 25th percentile; horizontal bar within box, median; open black circle in box: mean; upper bar outside box, 90th percentile; lower bar outside box, 10th percentile. Small red dots present raw data.

## 3. Results and Discussion

### 3.1. Single Z-Disc Removal Leads to Lowered Force Generation in Cardiomyocytes

**Figure 2:**
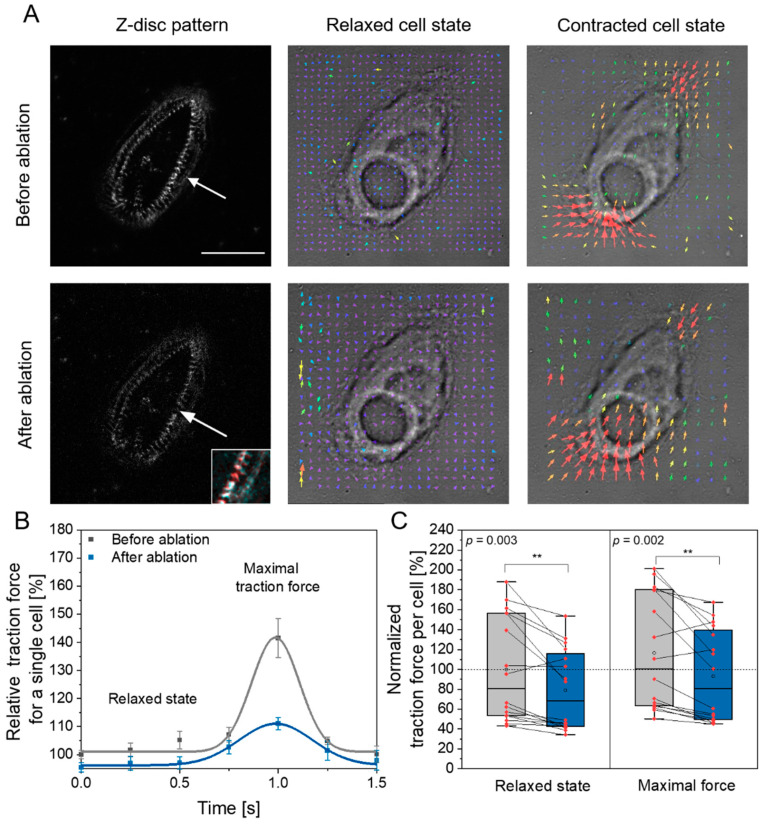
Traction force microscopy of hPSC-derived CMs before and after laser ablation of a single Z-disc. (**A**) Microscopic presentation of the same cell before and after ablation of a single Z-disc (arrow) in the relaxed and the contracted state. Left images show the Z-disc pattern in a relaxed cell, the small magnified image in the right corner highlights the removed Z-disc in a colored overlay of the pattern before (red) and after (cyan) Z-disc removal. Right images present force vectors from traction force microscopy in relaxed and contracted state. Scale bar 50 µm. (**B**) Outline of relative force determined via traction force microscopy. The measured curves were obtained by averaging twitches of a single cell and are normalized to the force of the relaxed state before ablation. A lower magnitude of the maximum contractile force after ablation is visible. (**C**) Analysis of the force produced by each cell before and after Z-disc removal in the relaxed and state of maximal force. Data are normalized to the mean maximal force before laser ablation (mean of grey box (open circle)). Data are based on 16 cells and at least 10 contractions per cell were analyzed. A significant lower force (**) was observed after ablation of a Z-disc.

In the first set of experiments, we were interested in whether ablation of a single Z-disc leads to changes in cellular force generation. Single cell traction force microscopy has been applied in CMs before, for instance, to determine the influence of cell shape via micro patterning and of maturation time [40]. In the study of Wheelwright et al. [40], cell length and maturation time were connected to sarcomeric cytoskeletal organization and correlated positively with traction force generation in the study. Therefore, a potential relation is possible between loss of Z-disc and the ability to generate traction forces. In our previous study, we observed sarcomere shortening of 6–7% to the initial length after laser Z-disc ablation in neonatal rat cardiomyocytes and hypothesized force generation was not significantly altered after micro-damage [30]. However, other earlier studies revealed that sarcomeric lesions are non-contractile and can slack myofibrils [41]. Therefore, we used traction force microscopy to analyze whether CMs exhibit less traction forces after Z-disc removal (see Figure 2).

A typical cardiomyocyte twitch, which we observed before and after laser ablation, is depicted in Figure 2B. In particular, we were interested in the traction force generated by each CM. Analysis of this force in all CMs before and directly after ablation showed that cells which underwent Z-disc removal generated around 20% less maximal traction forces (Figure 2C, right). This change was significant (*p* = 0.002, two sided *t*-test). The traction force loss in the relaxed state was of similar magnitude and also significant (Figure 2C, left, *p* = 0.003, two sided *t*-test). When we plotted the maximum generated force against the cell size before and after ablation and against the aspect ratio (see Appendix B), it was highlighted that these did not change as strongly as force generation. Reduced force generation was also observed in a recent study, by which CMs on hydrogels where physically stretched and hence myofibrils randomly ruptured [42].

The number of spontaneous contractions per time after ablation was on average 18% increased compared to the state before laser ablation, however, this change was not significant (*p* = 0.14, paired *t*-test). This is in agreement with studies from Leber et al., where contractility is preserved after sarcomere damage [43]. In addition, in one of our previous studies, we also observed no changes in calcium homeostasis [31] as well as constant contraction time, period and relaxation duration after single Z-disc removal [30,31]. Recently, Ufford et al. demonstrated that the myofibrillar structure and density correlates with hPSC-CMs contractile function using traction force microscopy [44]. This is in good agreement with our observations. Laser ablation of single Z-discs leads to myofibrillar rearrangement [30,31] and thus to changes in force generation. Additionally, the remodeling of the sarcomeric cytoskeleton might consume energy, which is not available for contraction output.

The hPSC-derived CMs applied in our study design have an immature sarcomere organization, which results in a non-parallel pattern of the myofibrils, in contrast to adult cardiomyocytes. This probably leads to lower traction forces and might also affect the measured magnitudes because of the force components in all axial directions. In agreement to our study, the sensitivity of traction force microscopy to detect changes in the force generation has also been demonstrated in genetic hPSC-derived CMs variants [45]. In future studies, it could be of high interest to seed cells in defined patterns and investigate both nanosurgery and its combination with traction force microscopy in those aligned CMs. Additionally, high-frame rate measurements of both traction forces and calcium homeostasis might complete our results and generate a holistic understanding of force development after nanosurgery. Our measurements were limited by the available frame rate of four FPS, which we tried to balance by averaging of several twitches.

### 3.2. Z-Disc Removal Is Associated with Gene Expression Changes in Markers for Cell Stress, Injury, and Sarcomeric Cytoskeleton Remodeling

**Figure 3:**
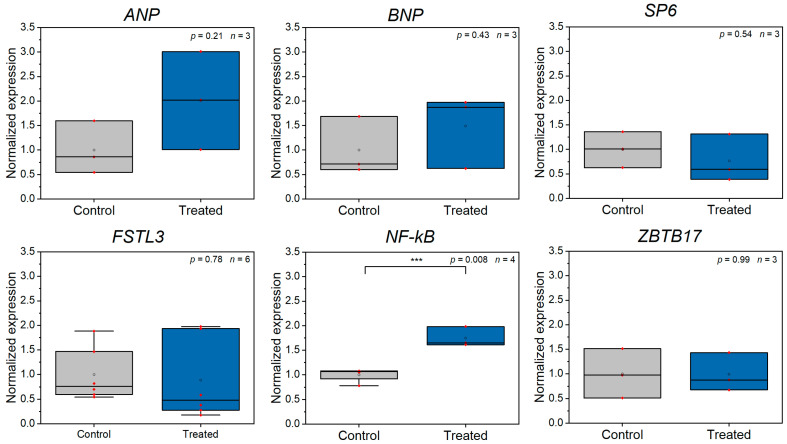
RT-qPCR analysis of stress-related gene expression comparing control CMs and CMs, which were laser manipulated (*n* ≥ 3 experiments). *NF-kB* was significantly upregulated (***) after Z-disc removal. All data were calculated according to Taylor et al. [39] and are normalized to the mean of the control group, small red dots present raw data.

We were further interested in whether Z-disc ablation results in any changes in gene expression. Based on our own observation on sarcomere repair and studies by Reibeiro et al. or Leber et al., revealing myofibril repair within about 4 h, we used this time point for gene expression analysis [42,43]. We hypothesized that the first cellular response to Z-disc damage would involve the activation of cell stress related genes. Further, also the laser manipulation itself might already induce activation of stress related genes. Therefore, we conceived a panel of genes, which are related to cellular stress or cardiomyocyte specific stress (e.g., stretch) and tested their expression after laser ablation of a Z-disc in comparison to untreated control cells (see Appendix A and Figure 3).

*ANP*, *BNP*, *SP6* and *FSTL3* are strongly connected to cell stretch or CM survival [46,47,48]. We found no up- or downregulation of these genes upon Z-disc removal. *ZBTB17*, a gene connected to cardiomyocyte survival was also not affected [49]. Only in the case of *NF-kB* was a significant upregulation found (*p* = 0.008, two-sided *t*-test). All other stress related genes were not significantly upregulated.

The application of femtosecond laser-based nanosurgery might also induce further downstream effects, which would, in particular, impact stress genes. Although our previous studies have shown that no measurable production of reactive oxygen species in the Z-disc region occurs [30] and damage is highly localized [31], we cannot fully exclude this. Unfortunately, this can also not be further elucidated, due to the strong dependence of the process on the cell type and the targeted structure [50].

**Figure 4:**
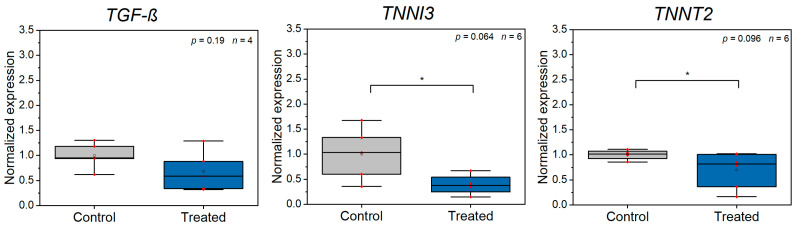
RT-qPCR analysis of injury and contraction related gene expression comparing control CMs and CMs, which were laser manipulated (*n* ≥ 3 experiments). TNNI3 and TNNT2 were significantly (*) downregulated. All data were calculated according to Taylor et al. [39] and are normalized to the mean of the control group, small red dots present raw data.

Next, we were interested in whether injury and contraction specific genes are affected by Z-disc loss (see Appendix A and Figure 4). We analyzed the contraction related genes TNNT2 and TNNI3. Further, we analyzed TGF-ß, which is, for instance, involved in several intracellular repair processes [51]. TNNT2 and TNNI3 were significantly downregulated (TNNT2: *p* = 0.096, TNNI3: *p* = 0.064, two-sided *t*-test). TNNT2 and TNNI3 have a close interdependence and Sehnert et al. demonstrated that loss of TNNT2 is associated with a significant reduction in TNNI3 [51]. As both are central for contraction of myocytes, the reduction of expression might also be related to our observation of lower force generation after Z-disc ablation, while the number of contractions remained unaffected.

**Figure 5:**
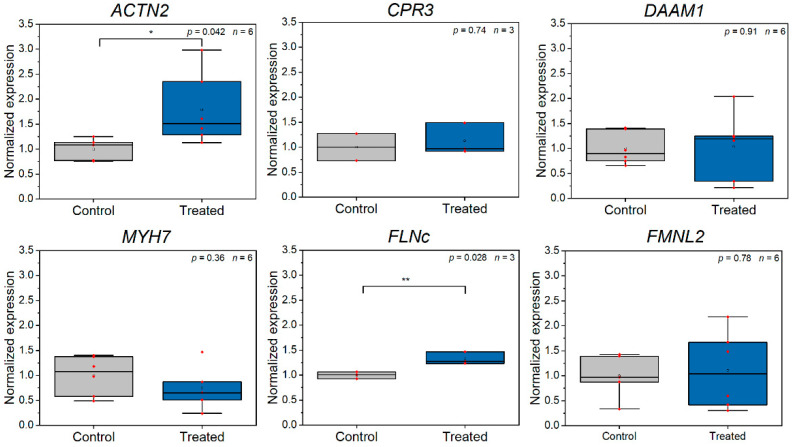
RT-qPCR analysis of sarcomeric cytoskeleton related gene expression comparing control CMs and CMs, which were laser manipulated (*n* ≥ 3 experiments). ACTN2 and FLNc were significantly (ACTN: *, FLNC: **) upregulated. All data were calculated according to Taylor et al. [39] and are normalized to the mean of the control group, small red dots present raw data.

Additionally, we were interested in genes, which are specific to the sarcomeric cytoskeleton (see Appendix A and Figure 5). We found a significant upregulation of ACTN2 (*p* = 0.042, two-sided *t*-test), which codes for the major component of the Z-disc, α-actinin [13]. We assume that this increase of expression is predictable, as the loss of a Z-disc is usually compensated by formation of a novel Z-disc [30,31]. Filamin-C (FLNc) was also significantly upregulated (*p* = 0.028, two-sided *t*-test). Leber et al. and Orfanos et al. analyzed the contribution of FLNc during repair of damaged myofibrils and observed a recruitment of FLNc to sarcomeric lesions [41,43]. Consequently, the upregulation of FLNc might be explainable by the requirement of novel FLNc protein in later processes. However, Leber et al. also observed that a block of protein biosynthesis does not hinder repair of damaged myofibrils [43]. Therefore, new synthesized FLNc might not be required to repair small numbers of damaged Z-discs. CPR3, DAAM1 and FMNL2, which are all involved in maintenance of the cardiomyocyte cytoskeleton and myofibrillogenesis [52,53,54], were not significantly affected by Z-disc removal. Additionally, MYH7 [55], which codes for the major protein of the thick filament, was unaffected. We assume that the damage is not sufficient to result in changes in these expression levels.

## 4. Conclusions

The precise manipulation of cytoskeletal elements via a femtosecond laser system enables a better understanding of the function and regeneration of the sarcomeric cytoskeleton in cardiomyocytes. We have extended our previous analysis on Z-disc removal in cardiomyocytes via analysis of traction forces and gene expression determination. Both techniques required a careful establishment, in particular, due to the lower number of cells which can be targeted via femtosecond laser manipulation. To our best knowledge, the effect of such micro-damage on gene expression has not been analyzed before. We found elevated expression levels of ACTN2 and FLNc, which is in good agreement with an early remodeling of the sarcomeric cytoskeleton. Moreover, the observed changes in contraction forces are in agreement with the downregulation of contraction-related genes. In an in vivo model, the changes in gene expression and force development might be connected to a process, which can be called sarcomeroptosis [14]. Sarcomeroptosis is a form of apoptosis, which implies the transfer of sarcomere activity into pro-survival pathways. Heart failure and pressure overload-induced cardiac hypertrophy can be experimentally modeled via transverse aortic constriction, which might be followed by sarcomeroptosis in CMs. This might especially involve the protein telethonin [14]. However, for instance, NF-kB is also regarded as anti-apoptotic, which might point to a link between our observations and the form of sarcomeroptosis in vivo.

## Figures and Tables

**Figure A1:**
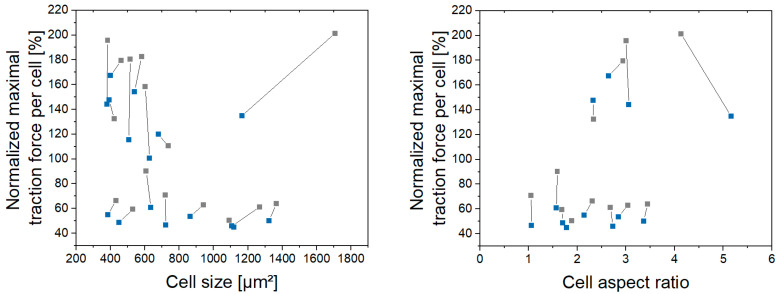
Maximal measured traction force in relation to cell size and aspect ratio (long to short axis) before (grey dot) and after (blue dot) laser ablation for each cell. The size and aspect ratio do not change strongly, which is indicated by the nearly vertical lines.

## Data Availability

The authors confirm that the data supporting the findings of this study are available within the article.

## Appendix A

**Table A1:** List of genes analyzed in RT-qPCR.

ID	Name	FW Primer 5′-3′	REV Primer 5′-3′	Function	Ref.
**Genes related to cellular stress (general stress and cardiomyocyte specific, for example, strech induced)**					
*ANP*	Natriuretic peptide A	CAGGATGGACAGGATTGGA	TGTCCTCCCTGGCTGTTATC	Strong connection to mechanical stretch of cardiomyocytes.	[43]
*BNP*	Natriuretic peptide B	TTGGAAACGTCCGGGTTAC	GGACTTCCAGACACCTGTGG	Strong connection to mechanical stretch of cardiomyocytes.	[43]
*SP6*	Sp6 transcription factor	GAGGACCTGGAAAGCGACAG	GATGAAGGTCCCACCACGAG	Strong connection to mechanical stretch of cardiomyocytes.	[44]
*FSTL3*	Follistatin like 3	CACCCGGGGAACAAGATCAA	GTCGCACGAATCTTTGCAGG	Strong connection to mechanical stretch of cardiomyocytes.	[44]
*NF-ΚB*	Nuclear factor kappa B subunit 1	AATTAACGGCGACAATCTGGAA	ACTTCACAAGCATAGCCATCAG	General regulator of stress reponse.	[46]
*ZBTB17*	Zinc finger and BTB domain containing 17	GTGTGATGTGCGGTAAGGC	TGGACTGGACGAATCTCTTGC	Can protect cardiomyocytes from apoptosis.	
**Genes related to cardiac injury**					
*TGF-Β*	Transforming growth factor beta	AAGATGACCGCTCTGACATCA	CTTATAGACCTCAGCAAAGCGAC	General marker of injury.	
*TNNI3*	Troponin I, cardiac muscle	CCAACTACCGCGCTTATGC	CTCGCTCCAGCTCTTGCTTT	Involved in sarcomere assembly and contraction. Marker of myocardial injury.	[47]
*TNNT2*	Cardiac muscle troponin T	TGGAGGCAGAGAAGTTCGAC	CCTGTTTCGGAGAACATTGAT	Involved in sarcomere assembly and contraction. Marker of myocardial injury.	[47]
**Genes related to sarcomeric cytoskeletal organization**					
*ACTN2*	Actinin alpha 2	CAAACCTGACCGGGGAAAAAT	CTGAATAGCAAAGCGAAGGATGA	Located at the Z-disc, cross-links actin and titin filaments.	[13]
*CSRP3*	Cysteine and glycine-rich protein 3	CCTGTGAAAAGACCGTCTACC	GTCGTGCTGTCAAGAGCCT	Involved in establishment and maintenance of the cardiomyocyte cytoskeleton.	[50]
*DAAM1*	Disheveled associated activator of morphogenesis 1	AGTATGCCAGCGAAAGGACC	TTCATCTCGATACCGCCCAGT	Located at the Z-disc. Regulates filamentous actin assembly.	[48]
*MYH7*	Myosin heavy chain 7	CGAAGGGCTTGAATGAGGAGT	TCCTCCCAAGGAGCTGTTAC	Major protein of the thick filament.	[51]
*FLNC*	Filamin C	CTGGGCGATGAGACAGACG	CGGATGGAACTTGCGGTA	Is involved in early stages of myofibrillar remodeling and repair.	[41]
*FMNL2*	Formin like 2 encodes a formin-related protein	GCTATGAACCTACCTCCTGACA	AACACGCCGTCTGAATTTCTT	Required for myofibrillogenesis.	[49]
**Housekeeper genes**					
*ATP5F1*	ATPase subunit b	AGGTCCAGGGGTATTGCAG	TCCTCAGGGATCAGTCCATAAC		
*GAPDH*	Glyceraldehyde-3-phosphate dehydrogenase	AGCCACATCGCTCAGACACC	GTACTCAGCGCCAGCATCG		
